# Center of Pressure in the Paws of Clinically Sound Dogs in Comparison with Orthopedically Diseased Dogs

**DOI:** 10.3390/ani10081366

**Published:** 2020-08-06

**Authors:** Bianca Reicher, Alexander Tichy, Barbara Bockstahler

**Affiliations:** 1 Department of Companion Animals and Horses, University Clinic for Small Animals, Small Animal Surgery, Section of Physical Therapy, University of Veterinary Medicine, 1210 Vienna, Austria; barbara.bockstahler@vetmeduni.ac.at; 2 Department of Biomedical Sciences, Platform of Bioinformatics and Biostatistics, University of Veterinary Medicine, 1210 Vienna, Austria; alexander.tichy@vetmeduni.ac.at

**Keywords:** balance, center of pressure, dog, gait analysis, osteoarthritis

## Abstract

**Simple Summary:**

The analysis of the center of pressure is an important tool that allows conclusions to be drawn about the body balance of a patient. This balance can be altered by orthopedic or neurological diseases. To date, there are few data on the center of pressure in the paws of walking dogs. This study aimed to show the compensatory changes in the center of pressure parameters within the paw during stance-phase in the walking dog, with data being collected non-invasively by walking dogs with osteoarthritis and healthy dogs (control group) over a pressure platform. Differences in the center of pressure parameters were found between the affected and non-affected limbs of the diseased dogs, but also in comparison to the corresponding limbs of sound dogs. It was shown that dogs with osteoarthritis show different compensatory changes in the center of pressure parameters depending on whether a front or a hind limb are affected. This can reflect a compensatory redistribution of the body mass as well as compensatory changes of body balance. Based on these observations, a deeper investigation of the center of pressure within the paws of dogs would be of interest and a diagnostic application could become possible in the future.

**Abstract:**

The center of pressure (COP) is recognized as a valuable tool for the assessment of orthopedic and neurologic disorders in humans. Relatively few studies are available in veterinary medicine, particularly concerning the COP in the individual paw. This study assessed the dynamic paw COP parameters during the stance phase of dogs with cox- or cubarthrosis (20 dogs each), as well as of 20 sound dogs. Data were obtained by walking over a pressure platform and analyzed within the diseased groups in comparison to the control group. Both diseased groups showed significant differences between the affected and non-affected limbs, but also in comparison to the reference limbs of sound animals. For coxathrosis, the primary increase was in the medio-lateral COP displacement and the COP area in both hind limbs. For cubarthrosis, the most prominent changes were an increase in the medio-lateral COP displacement in the ipsilateral hind limb and in the cranio-caudal COP displacement in the lame limb. Additionally, the COP area increased in both hind limbs. This can reflect a compensatory redistribution of the body mass as well as compensatory changes of body balance.

## 1. Introduction

In veterinary medicine, a wide range of applications for the evaluation of ground reaction forces can be found. In addition to research on the physiological movement patterns of various species [1,2], such evaluations serve as a valuable tool in the investigation of orthopedic and neurologic diseases of dogs [3,4] and enable new findings in therapy [5]. In human medicine, the measurement of the center of pressure (COP) is also frequently used to examine patients with orthopedic or neurological diseases [6,7,8,9].

The COP is described as the location where the instantaneous vector of the ground reaction forces acts. During ground contact, the position of the COP changes continuously, thus creating the COP path [9]. The evaluation of the COP is already being used as a way of obtaining information about the postural control of human patients. This is defined as the act of maintaining, achieving, or restoring a state of equilibrium during posture or activity [10]. If a patient has problems maintaining, achieving, or restoring a state of equilibrium, this implies a deficit in postural control, which may be caused by a variety of cognitive, sensory, or motor impairments. The displacement of the COP is an indirect measure of the functionality of postural control and thus a measure of a person’s ability to maintain balance. This natural sway of the COP can be measured using force plates and represented mathematically using a variety of parameters. This so-called posturography has proven to be a comprehensive tool for objective and sensitive analysis of postural control. It is therefore the gold standard for laboratory measurement of postural control in sound and diseased individuals [11]. Human orthopedics has shown that various COP parameters, such as COP speed and area, are altered in patients with osteoarthritis. This is usually attributed to reduced proprioception and muscle strength and instability of the joint.

One study from 2006 investigated the effect of gonarthrosis on the gait in humans. The results of static pedobarography showed a positive correlation between body COP sway length, width and the severity of gonarthrosis. Thus, it was shown that, with increasing severity of osteoarthritis, the impairment of balance also increases [8].

In 2018, another study investigated differences in balance between two stabilization techniques used in the treatment of degenerative changes of the ankle joint in humans. The data were collected by bipedal stance on a pedobarography plate and not only revealed that there are differences between the techniques used, but also that the patients balance could not reach normal values after either of the two procedures tested [7]. 

A study on postural balance in patients with osteoarthritis of the hip joint collected data of patients in a standing position. Numerous significant differences between the patients and a sound control group were found. Altered parameters included, among others, the sway path, the length of the COP path in the medial-lateral plane and mean COP velocity [12].

The long-term changes in gait pattern after talus fractures in humans have been investigated using gait analysis insoles. This method allowed the authors to collect data on the center of pressure of both feet while the participants were walking [13].

It should therefore be noted that there are studies in which the body COP [4,7,8,14,15] was investigated, while other studies focused on the COP of the individual foot (or paw) [13,16,17,18,19]. In addition, data concerning the COP have been obtained by static (standing) [4,7,8,14,15,16] or dynamic (locomotion) [16,17,18,19] measurements in various studies. 

In contrast to human medicine, to date, few studies have been conducted in veterinary medicine to evaluate the COP in sound animals and animals suffering from orthopedic diseases. One study investigated different body COP parameters and their correlation to different morphological features of sound horses. The data was collected during stance on two aligned force plates, so that data of all four limbs could be collected. It was found that some COP parameters (namely craniocaudal velocity and the calculated mediolateral frequency) are influenced by the size of the animal. For all other evaluated parameters, no size dependency could be found [15]. As has already been established in humans [20], the balance is not only influenced by the body’s inherent feedback systems but also by the sense of vision. To investigate the importance of the visual sense for postural stability, another study on horses was conducted in a similar manner. The results showed that, after blindfolding of sound horses, increased instability can be observed [21]. Another study looked at changes in body COP parameters in ponies with forelimb lameness compared to sound animals. The animals stood on a pressure plate with both forelimbs during data collection. It was found that the diseased animals had a larger COP sway according to various parameters (e.g., COP velocity and length) [14].

The body center of pressure variability in sound chondrodystrophoid dogs has been investigated by using an instrumented force-plate treadmill. By tracking a marker positioned in the interscapular area with infrared sensors, the movement of the body COP relative to this anatomical marker could be recorded. It was found that the COP was moving in a butterfly-like path during the gait cycle [22]. Another study with a similar design examined non-ambulatory dogs with thoracolumbar spinal cord injury. During the measurements, the hind limbs of these animals were supported with slings. It was shown that the COP in these animals is located more cranially and that there is more variability in both the craniocaudal and the left-to-right movement when compared to the results of the study on sound chondrodystrophoid dogs mentioned above [23].

Another study investigated the use of static posturography as an additional tool in the assessment of lameness. The data of sound and diseased dogs (unilateral elbow osteoarthritis) were collected while standing on a pressure platform. The obtained statokinesiogram showed not only a larger and more asymmetric body COP sway area in diseased animals compared to the sound control group, but also a significant reduction of the sway area after treatment with plasma rich in growth factors. Furthermore, a stabilogram revealed that the oscillations of the body COP were greater and/or more asymmetric in the lame dogs. Additionally, a graphic illustration of the pressure distribution in the paws showed not only a shift of the body COP towards the sound limb, but also a shift of the maximal pressure point and the limb COP. While the maximal pressure point shifted laterally in the sound limb of diseased animals, a cranial shift was found in the lame limb. The limb COP of the lame limb shifted craniomedially while it stayed centered in the sound limb of diseased animals [24].

A recent study investigated various parameters of posturography and dynamic pedobarography in dogs with osteoarthritis due to elbow dysplasia, as well as dogs with cranial cruciate ligament rupture and in a control group of sound animals. The data concerning the COP were collected during standing on a pressure plate with either both front limbs or hind limbs, depending on the location of the lameness. The evaluation of the obtained statokinesiograms and stabilograms showed significantly higher COP sway in the diseased groups compared to the control group. In the comparison of the two diseased groups, the animals with elbow disease showed even higher values in the statokinesiogram than those with cranial cruciate ligament rupture [4].

Another recently published study was, to our knowledge, the first to investigate the center of pressure path within the paw in dogs. The study focused on the differences between sound and lame limbs in dogs with unilateral lameness due to osteoarthritis of the elbow. The data concerning the COP path were obtained during walking over a pressure plate. All parameters concerning the COP path (for example the COP path length) showed significant differences between the lame and the sound forelimb. Additionally, a statokinesiogram was created during standing with both forelimbs on the pressure plate. The area of the statokinesiogram showed lower values in the sound limb and thus more instability in the diseased limb. The study concluded that COP characteristics can be used to differentiate between sound and diseased extremities [16].

Another recent study used a pressure platform to investigate the usefulness of pedobarographic parameters for the evaluation of dogs with osteoarthritis, in particular, before and after the administration of mavacoxib. Although data concerning COP sway and mediolateral imbalance were acquired during walking, they were not quantified. Nonetheless, the authors found clear evidence for postural adjustments and joint instability, as well as their improvement after treatment with mavacoxib [25].

In general, most studies measure the COP while standing. Although this is an excellent approach, this method can be somewhat difficult in dogs. It can sometimes be challenging to have dogs stand motionless for long periods of time - especially if they suffer from a painful joint disease. Most dogs tend to sit or lie down to relieve the painful limb. In this study, the COP path within the paws during the stance phase was therefore measured while the dogs were guided over a pressure plate. The present study, however, aims not only to compare lame and sound limbs within an animal, but also to compare them to the performance of sound animals. 

The hypothesis of this study is that a stance phase lameness caused by osteoarthritis leads to a compensatory change of the COP-path within the paws and thus parameters of the COP can be used to depict an osteoarthritis-related lameness. These compensatory changes should clearly differ between osteoarthritis of the hip joint and osteoarthritis of the elbow joint as they represent examples of hind limb and fore limb lameness.

## 2. Materials and Methods

### 2.1. Ethics

The data of the diseased dogs were widely collected within the routine clinical examinations at the Section for Physical Therapy and Rehabilitation of the University of Veterinary Medicine, Vienna. In addition, some measurement data from voluntary sound participants and data collected in previous studies were used. The data were all obtained using the same standardized measurement procedure.

The conduct of all these measurements was discussed and approved by the institutional ethics and animal welfare committee in accordance with Good Scientific Practice guidelines and national legislation (ETK-04/05/97/13, ETK-01/03/2017, ETK-09/12/2015, ETK-05/09/2016).

### 2.2. Animals and Inclusion Criteria

In total this study involved 63 client-owned dogs.

Study group 1 consisted of dogs with osteoarthritis of the hip. It included seven mixed breeds, three Golden Retrievers, two Rottweilers, two Labradors, two German Shepherds, one Tibetan Terrier, one Samoyed and one Appenzeller Mountain Dog, making a total of 19 dogs. The dogs had a mean body mass of 29.66 ± 11.89 kg and the mean age was 7.73 ± 3.67 years. The group included 13 male/male neutered and 6 female neutered animals.

Study group 2 consisted of dogs with osteoarthritis of the elbow. It included eight mixed breeds, five Golden Retrievers, three Labradors, one German Shepherd, one American Bulldog, one Newfoundland, one Magyar Vizsla, one Pointer, one English Cocker Spaniel, one Dachshund and one American Staffordshire Terrier, making a total of 24 dogs. The dogs had a mean body mass of 30.72 ± 10.10 kg and the mean age was 7.13 ± 0.89 years. The group included 14 male/male neutered and 10 female/female neutered animals.

The inclusion criteria for both study groups were a uni- or bilateral osteoarthritis in the respective joint, confirmed by an orthopaedical examination and by imaging techniques. Dogs were only included if they had clearly visible lameness in one front limb/hind limb, pain during manipulation of one elbow or hip joint during orthopedic examination and confirmed lameness as determined via ground reaction forces analysis on the pressure plate. This lameness had to be quantified by calculating a symmetry index (SI%). The inclusion threshold was an SI% of ≥ 3% in accordance with the threshold used for the sound control group. In addition, the animals in study group 1 (osteoarthritis of the hip) were not to exhibit any pain in or orthopedic impairment of the front limbs, and those in study group 2 (osteoarthritis of the elbow) were not to exhibit any pain in or orthopedic impairment of the hind limbs.

The control group consisted of sound dogs. It included five mixed breeds, four Golden Retrievers, two Rottweilers, one German Shepherd, one Labrador, one Dachshund, one Berger Blanc Suisse, one Beagle, one Doberman, one French Bulldog, one Setter, and one Dalmatian, making a total of 20 dogs. The dogs had a mean body mass of 27.14 ± 9.13 kg and the mean age was 4.4 ± 3.18 years. The group included 10 male/male neutered and 10 female/female neutered animals. All sound dogs for the control group were examined orthopedically and neurologically by a clinician and found to be free of lameness and pain. To also exclude dogs with undetected problems that affect the gait, only dogs with SI% < 3% [26,27] were included in this group. If available, animals were chosen which resembled the dogs of study group 1 and 2 in body type [27].

### 2.3. Equipment

A pressure distribution measurement platform (FDM Type 2) made by Zebris Medical GmbH (Allgäu, Germany) was used to record the ground reaction forces. This platform is equipped with 15,360 sensors covering an area of 203 × 54.2 cm working with a measuring frequency of 100 Hz. The measurement procedure was filmed with a camera (Panasonic NV-MX500) and data were gathered using WinFDM software (v1.2.2, Zebris Medical).

The measurement platform was positioned in the middle of a 7-meter long walkway. A flat running surface was created by means of adjacent chipboards. Chipboard and platform were covered with a 2-mm-thick rubber mat to prevent visual irritation of the participants and slipping on the smooth surface. 

### 2.4. Measurement Procedure

Before measurement, dog and owner were given sufficient time to explore the measuring laboratory. After the animal was allowed to move freely throughout the room, it was led over the platform several times on a leash at walking speed until an even gait pattern was achieved. The dogs were, as is usual in Austria, led on the left side of the handler. This corresponds to the position in which the dogs are led by the owner in everyday life. During the following measuring procedure, the animal was led over the platform in a walking gait in the same manner until a minimum of 5 valid consecutive steps were recorded. A pass was considered valid if the dog moved at a steady pace, kept his head straight, and did not pull on the leash.

### 2.5. Data Analysis

The data was subsequently analyzed with the custom software Pressure Analyzer (Michael Schwanda, version 3.0.6344.26760). With the help of the recorded video the valid passes were identified. The individual footprints recorded during the valid passes were manually assigned to the corresponding extremity.

In study group 1, the hind limb that was less loaded was referred to as the lame limb (LA). To enable a statistical analysis, for study group 1 the lame limb (LA) was always compared to the right hind limb (RH) of the control group. Therefore, it follows that the contralateral limb (CL) was compared to the left hind limb (LH) of the control group, the ipsilateral limb (IL) was compared to the right front limb (RF) of the control group, and the diagonal limb (DI) was compared to the left front limb (LF) of the control group. For study group 2 on the other hand, the front limb that was less loaded was referred to as the lame limb (LA). Thus, in study group 2, the lame limb (LA) was always compared to the right front limb (RF) of the control group, the contralateral limb (CL) was compared to the left front limb (LF) of the control group, the ipsilateral limb (IL) was compared to the right hind limb (RH) of the control group, and the diagonal limb (DI) was compared to the left hind limb (LH) of the control group.

### 2.6. Parameters under Investigation

Investigated parameters were speed (m/s) and acceleration (m/s^2^), which were both calculated from the ground reaction force parameters of the left forelimb.

The peak vertical force (PFz, N) and the vertical impulse (IFz, Ns) of each limb were measured and then normalized to the total force (PFz%; IFz%) by applying the following equation to each limb:

(1) $$XFzLF\left(\%\right)=\frac{XFzFL\;}{\left(XFzFL+XFzFR+XFzHL+XFzHR\right)}*100$$

where XFz = mean value of PFz or IFz; LF: left front limb; FR: right front limb; HL: left hind limb; HR: right hind limb.

Furthermore, a symmetry index expressed as a percentage (SI%) was calculated for both parameters according to the following equation, modified from Budsberg et al. [28]:

(2) $$SIXFz\;\left(\%\right)=abs\left(\frac{\left(XFzLF-XFzRF\right)}{\left(XFzLF+XFzRF\right)}\right)*100$$

where SIXFz = SI of PFz or IFz; XFz = mean value of PFz or IFz; LF = left front limb; RF = right front limb.

For the calculation of the SI% of the hind limbs, the equation was applied in the same fashion.

Another parameter under investigation was the time at which the PFz is reached during the stance phase. This was expressed as a percentage of the stance phase duration (TPFz%). The stance phase duration was also investigated and was expressed as percentage of the total stance phase duration (SPD%).

**Figure 1:**
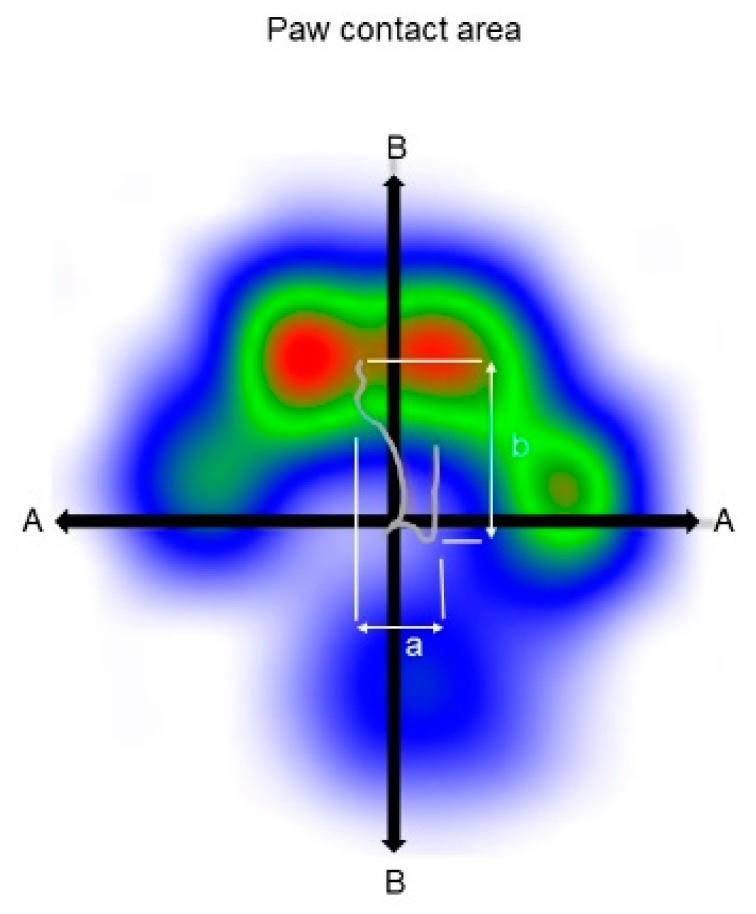
Paw contact area with COP path. The mediolateral (**a**) and craniocaudal (**b**) displacement of the center of pressure were expressed as a percentage of A and B, respectively. a = mediolateral displacement of COP; b = craniocaudal displacement of COP; A = maximum width of paw contact area; B = maximum length of paw contact area.

The following COP parameters were evaluated for the stance phases of each paw:

- Medio-lateral and cranio-caudal COP displacement: These parameters are the differences between the maximum positive and negative COP values along the cranio-caudal and medio-lateral axes, respectively [11,17,18]. The medio-lateral displacement was normalized to the maximum width of the paw contact area (COPmed-lat%) and the cranio-caudal displacement to the maximum length of the paw contact area (COPcran-caud%) as displayed in Figure 1.
- COP-Area: The COP area is a measurement of the area encompassed by the boundaries of the COP movement [11]. It was normalized to the paw contact area and expressed as a percentage (COP-Area%).
- COP-Velocity: The COP velocity is the mean speed of the movement of the COP (COP-Velocity, mm/s).
- COP-Radius: This is the mean distance of all COP points to the center point of all COP points. This parameter was also normalized to the paw contact area and given as a percentage (COP-Radius%).

### 2.7. Statistical Analysis

Statistical analyses were performed using IBM SPSS v24. The normal distribution of the parameters was proven by the Kolmogorov–Smirnov test. Linear mixed effects models were used to analyze the differences between groups, and hips, or elbows and limbs, where the factor hip or elbow and limb was treated as a repeated measurement. Post hoc tests were performed using Sidak’s alpha correction procedure. For all analyses, a *p*-value below 5% (*p* < 0.05) was seen as significant.

## 3. Results

The detailed results are discussed below separately for the two study groups. Additionally, a list of all *p*-values not explicitly mentioned in the text is given in Appendix A and an overview of the results of study group 1 and 2 is given in Appendix B.

### 3.1. Detailed Results of Study Group 1 (Coxarthrosis)

Study group 1 and control group differed significantly in age, *p* = 0.006) but not in body mass. The speed of the dogs with osteoarthritis of the hip (1.00 ± 0.15 m/s) was comparable (no significant difference) to the speed of the control group (1.11 ± 0.21, *p* = 0.10). The acceleration of the diseased dogs (0.03 ± 0.07m/s^2^) also did not show a significant difference when compared to the control group (0.00 ± 0.6 m/s^2^, *p* = 0.16). The SI% of the front limbs of sound dogs were 0.69 ± 0.62% for PFz and 1.02 ± 0.76% for IFz, while the SI% for PFz of dogs with osteoarthritis of the hip was 1.75 ± 2.31% and the SI% for IFz was 4.01 ± 3.70%. These parameters did not show any significant differences (*p* = 0.91 and *p* = 0.48) between the groups. The SI% of the hind limbs of sound dogs were 1.09 ± 0.70% for PFz and 1.29 ± 0.87% for IFz, while those of dogs with osteoarthritis of the hip were 5.72 ± 4.17% for PFz and 6.94 ± 4.73% for IFz. This resulted in a significant difference in the hind limbs of the SI% for PFz (*p* = 0.001) and for IFz (*p* = 0.001) between the groups.

**Figure 2:**
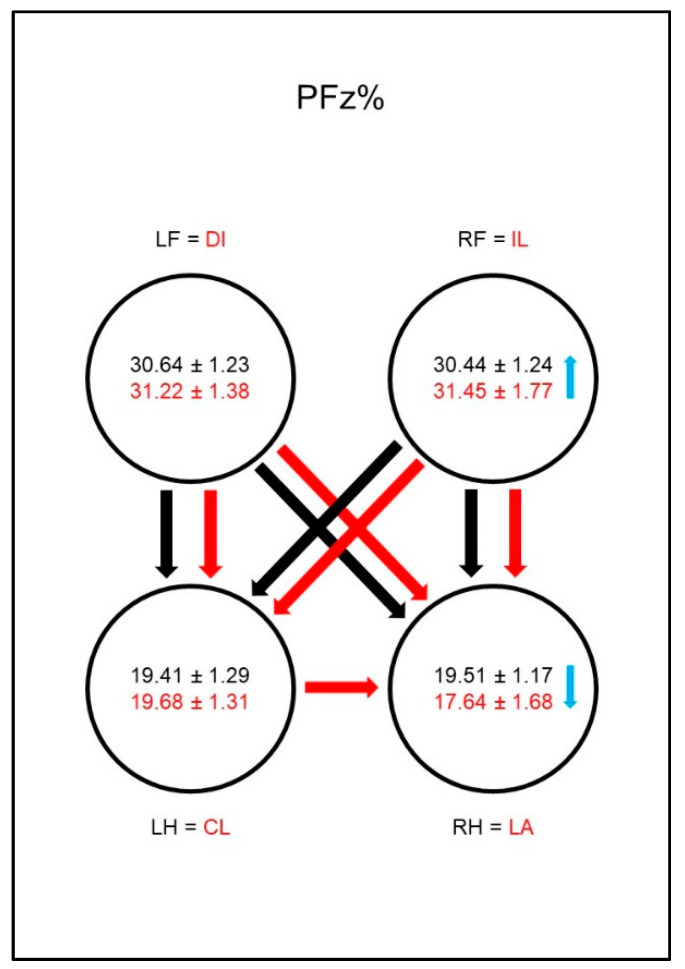
Peak vertical force as a percentage of total force (PFz%) in study group 1 and control group. Black writing and arrows refer to the control group, red writing and arrows refer to the study group. Arrows between the circles indicate significant differences between the individual paws within the respective group. The tip of the arrow always marks the lower value. For example, the lame limb/right hindlimb had a significantly lower PFz% than both front limbs. Blue arrows within the circles mark significant differences in the comparison between control group and study group. The direction of the blue arrows indicates whether an increase (up) or decrease (down) of the values has been observed in the study group. For example, the lame limb of the study group showed a significant decrease in PFz% compared to the right hind limb of the control group. Outlined blank arrows mark differences just outside the significance limit. LF = left front limb, RF = right front limb, LH = left hind limb, RH=right hind limb, LA = lame limb, DI = diagonal limb, IL = ipsilateral limb, CL = contralateral limb.

**Figure 3:**
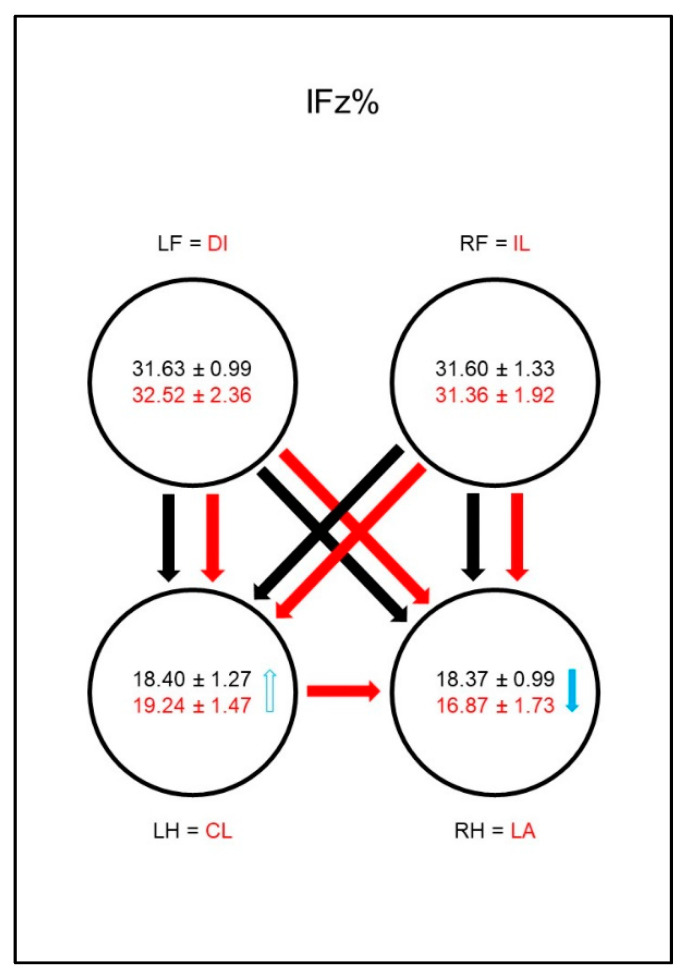
Vertical impulse as a percentage of total force (IFz%) in study group 1 and control group. For detailed explanation and abbreviations refer to Figure 2 (page 8).

The forelimbs showed significantly higher values for PFz% (Figure 2) and for IFz% (Figure 3) than the hind limbs in both sound and diseased animals. While in these parameters no differences between the contralateral limbs were found in sound animals, in dogs with osteoarthritis of the hip, these values were significantly lower in the lame limb than in the contralateral hind limb. This could also be observed in the group comparison: the diseased animals showed significantly lower values for PFz% and IFz% of the lame limb than the reference limb of sound dogs. PFz% was also significantly higher in the ipsilateral front limb than in the reference limb of sound dogs. The IFz% in the contralateral hind limb was higher in dogs with osteoarthritis of the hip than in the reference limb of sound dogs, but the significance limit was narrowly missed.

**Figure 4:**
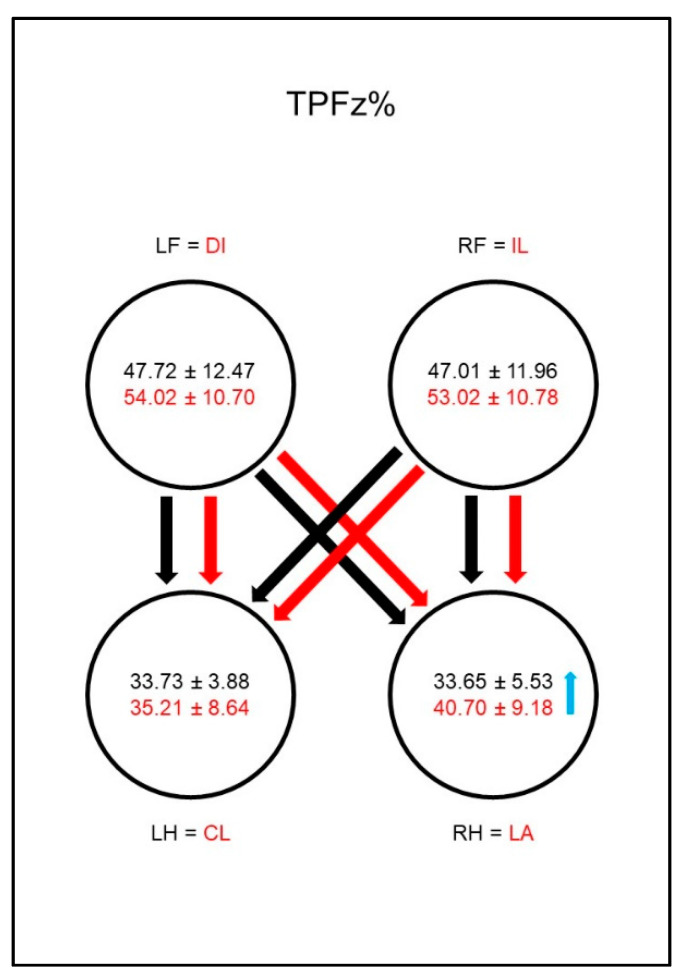
Time to PFz as a percentage of stance phase duration (TPFz%) in study group 1 and control group. For detailed explanation and abbreviations refer to Figure 2.

The PFz occurred earlier (TPFz%, Figure 4) in both groups in the hind limbs than in the front limbs, and there was no difference between the contralateral limb pairs. However, the group comparison showed a significantly later occurrence of PFz in the lame limb than in the reference limb of sound dogs.

**Figure 5:**
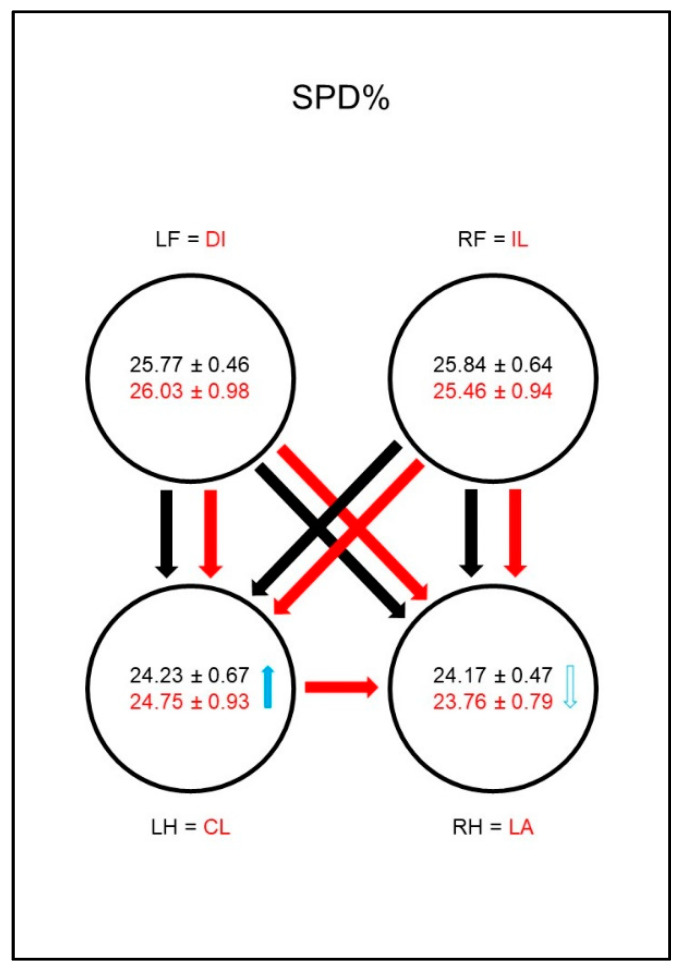
Stance phase duration as a percentage of total stance phase duration (SPD%) in study group 1 and control group. For detailed explanation and abbreviations refer to Figure 2.

The duration of the stance phase (SPD%, Figure 5) was significantly shorter in the sound animals in the hind limbs than in the front limbs. There was no difference between the contralateral limbs in this group. The difference between the front limbs and the hind limbs persisted in the diseased animals; in addition, there was a significantly shorter SPD% of the lame limb compared to the contralateral hind limb. The group comparison showed a significantly longer SPD% in the contralateral hind limb compared to the reference limb of the sound animals. Additionally, a shorter SPD% in the lame limb compared to the reference limb could be found, even though the significance limit was narrowly missed.

**Figure 6:**
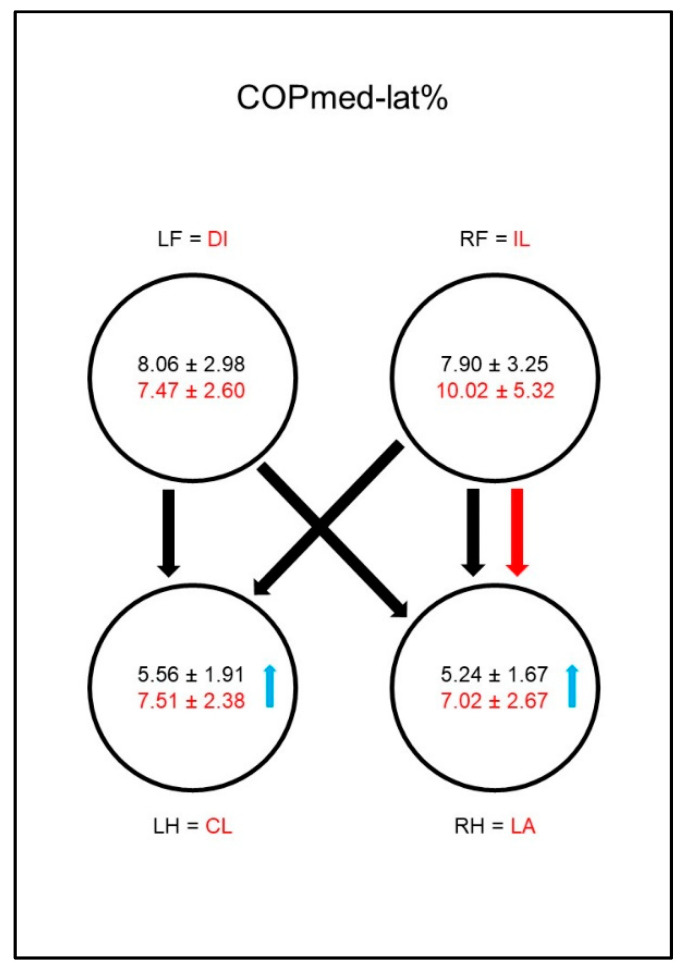
Medio–lateral displacement of the center of pressure as a percentage of maximum width of paw contact area (COPmed-lat%) in study group 1 and control group. For detailed explanation and abbreviations refer to Figure 2.

In sound dogs the mediolateral displacement of the COP (COPmed-lat%, Figure 6) was significantly smaller in both hind limbs than in the front limbs. In animals with coxarthrosis, the mediolateral COP displacement of both hind limbs was increased, resulting in a significant difference to the reference limbs of sound animals. This led to a vanishing of most of the significant differences between the paws in the diseased animals. The significant difference between the ipsilateral front limb and the lame limb was the only one that, due to a (not significantly) increased mediolateral displacement in the ipsilateral front limb, could still be observed.

**Figure 7:**
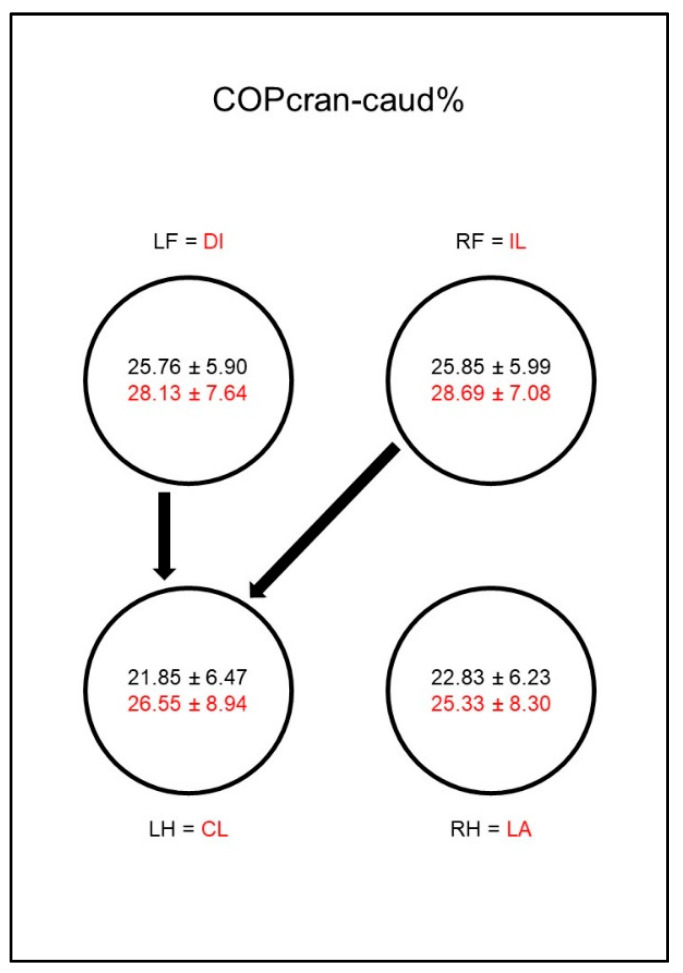
Cranio-caudal displacement of the center of pressure as a percentage of maximum length of paw contact area (COPcran-caud%) in study group 1 and control group. For detailed explanation and abbreviations refer to Figure 2.

Concerning the craniocaudal displacement of the COP (COPcran-caud%, Figure 7), the results were not consistent. In sound animals, the values in the hind limbs were, in general, lower than in the front limbs, but this was only significant when comparing the left hind limb with the front limbs, whereas it was not significant in the right hind limb. In the diseased animals, no differences to the other limbs could be shown, which was due to a relative increase of the craniocaudal displacement in all extremities. The group comparison of sound and diseased animals showed no differences.

**Figure 8:**
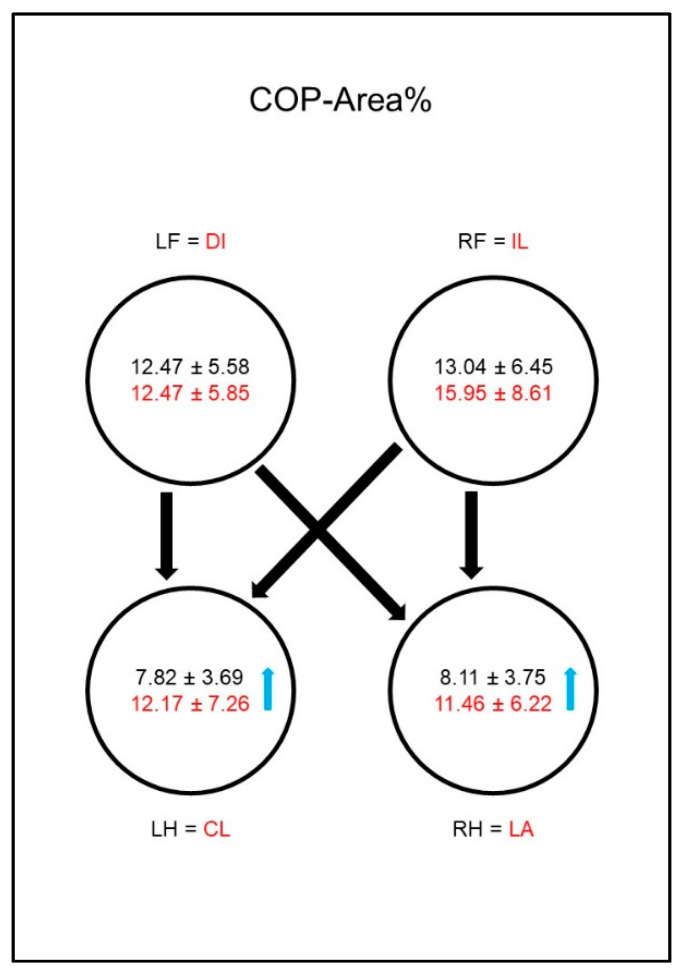
Area encompassed by the boundaries of the center of pressure movement as a percentage of paw contact area (COP-Area%) in study group 1 and control group. For detailed explanation and abbreviations refer to Figure 2.

The COP-Area% (Figure 8) in sound animals was significantly smaller in the hind limbs than in the front limbs, whereas in the diseased animals it was not. This was due to a significant increase in the COP-Area% in both hind limbs compared to sound dogs.

**Figure 9:**
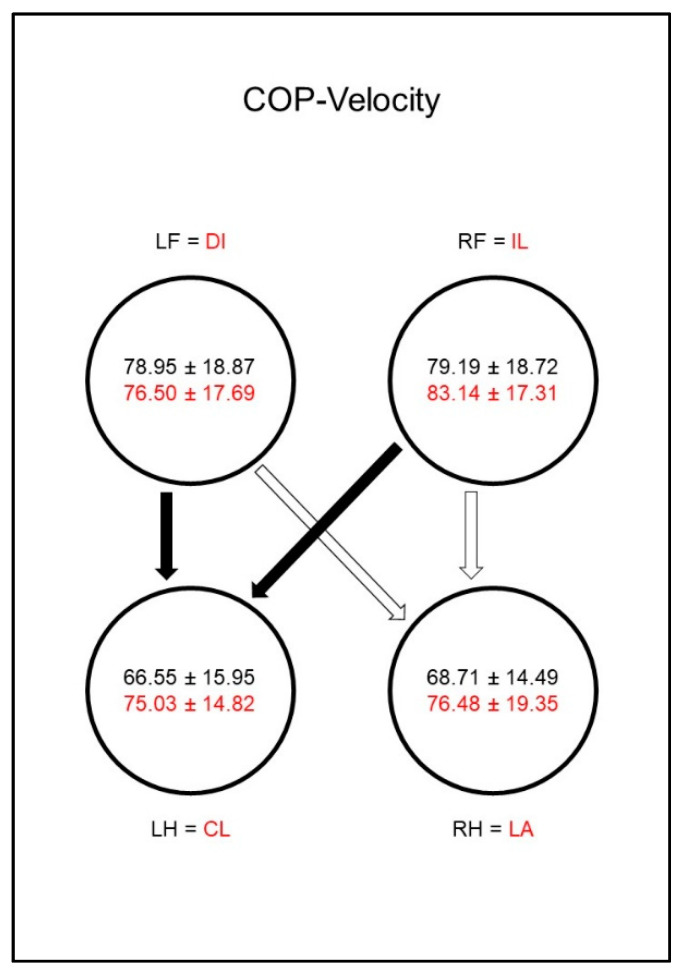
Speed of center of pressure movement in millimeters per second (COP-Velocity, mm/s) in study group 1 and control group. For detailed explanation and abbreviations refer to Figure 2.

The COP-Velocity (mm/s, Figure 9) in sound animals showed lower values in the hind limbs than in the front limbs, whereby the significance limit for the right hind limb was narrowly missed. In the diseased dogs there was a relative increase in the COP-Velocity (mm/s) of the hind limbs and the ipsilateral front limb, which led to a vanishing of the significant differences between the paws. However, the group comparison showed no significant differences.

**Figure 10:**
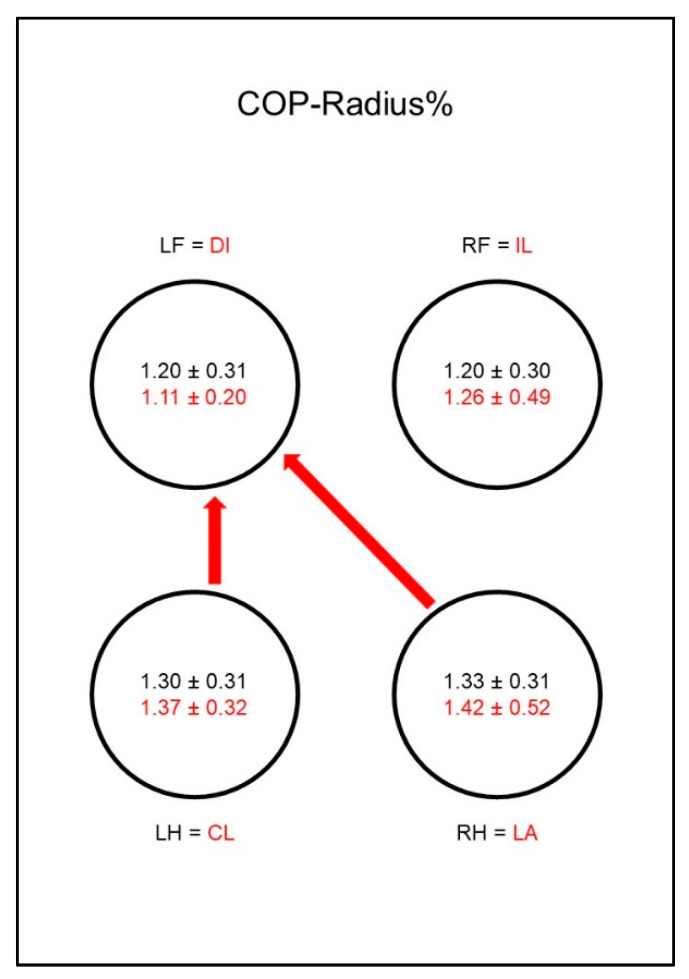
Mean distance of all center of pressure (COP) points to the center point of all COP points as a percentage of paw contact area (COP-Radius%) in study group 1 and control group. For detailed explanation and abbreviations refer to Figure 2.

A different situation could be seen with the COP-Radius% (Figure 10). While in sound animals it did not differ between the limbs, in sick animals there was a significant difference between both hind limbs and the diagonal front limb. This was due to a comparatively low COP-Radius% of the diagonal front limb and a relatively increased COP-Radius% in the hind limbs. However, there was no significant difference between the groups.

An overview of the significant differences in the COP parameters of study group 1 and the control group is displayed in Table 1.

### 3.2. Detailed Results of Study Group 2 (Cubarthrosis)

Study group 2 and control group differed significantly in age, *p* = 0.014) but not in body mass. The speed of the dogs with osteoarthritis of the elbow (0.98 ± 0.19 m/s) was comparable with the sound dogs (1.11 ± 0.21 m/s, *p* = 0.05), but in the acceleration of the diseased dogs (0.07 ± 0.09 m/s^2^) there was a significant difference to the sound dogs (0.00 ± 0.06 m/s^2^, *p* = 0.02). The SI% of the front limbs of sound dogs were 0.69 ± 0.62% for PFz and 1.02 ± 0.76% for IFz; while the SI% were 12.98 ± 8.83% for PFz and 15.71 ± 11.04% for IFz in dogs with osteoarthritis of the elbow. This resulted in a significant difference for the SI% in the front limbs for PFz (*p* < 0.001) and for IFz (*p* < 0.001). The SI% of the hind limbs of sound dogs were 1.09 ± 0.70% for PFz and 1.29 ± 0.87% for IFz, those of dogs with osteoarthritis of the elbow were 5.08 ± 5.06% for PFz and 5.59 ± 6.19% for IFz. This resulted in a significant difference of the SI% in the hind limbs for PFz (*p* = 0.004) and IFz (*p* = 0.01).

**Figure 11:**
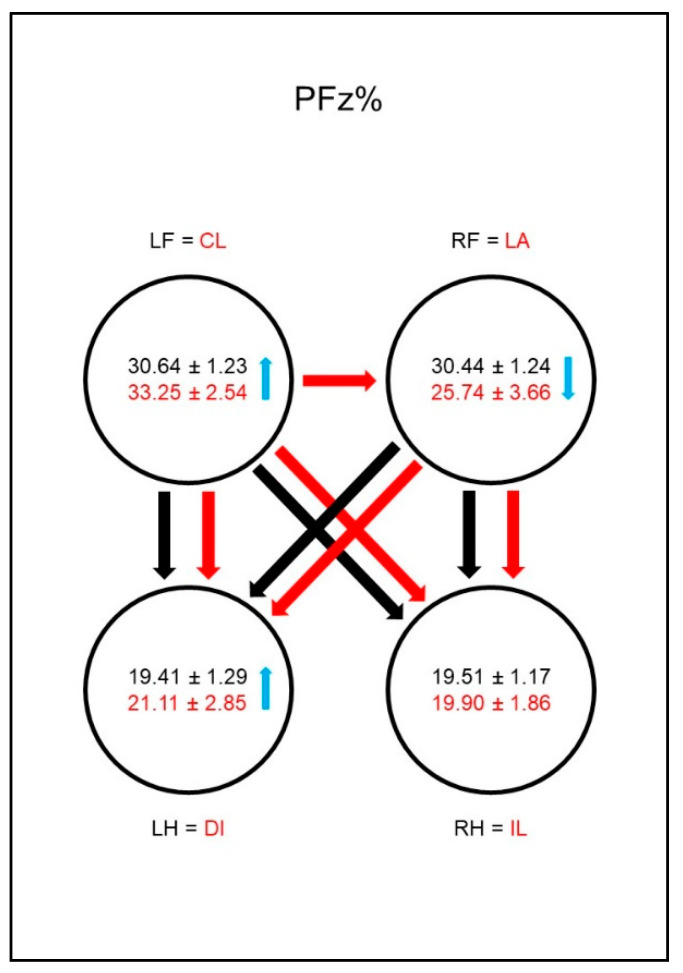
Peak vertical force as a percentage of total force (PFz%) in study group 2 and control group. For detailed explanation and abbreviations refer to Figure 2.

**Figure 12:**
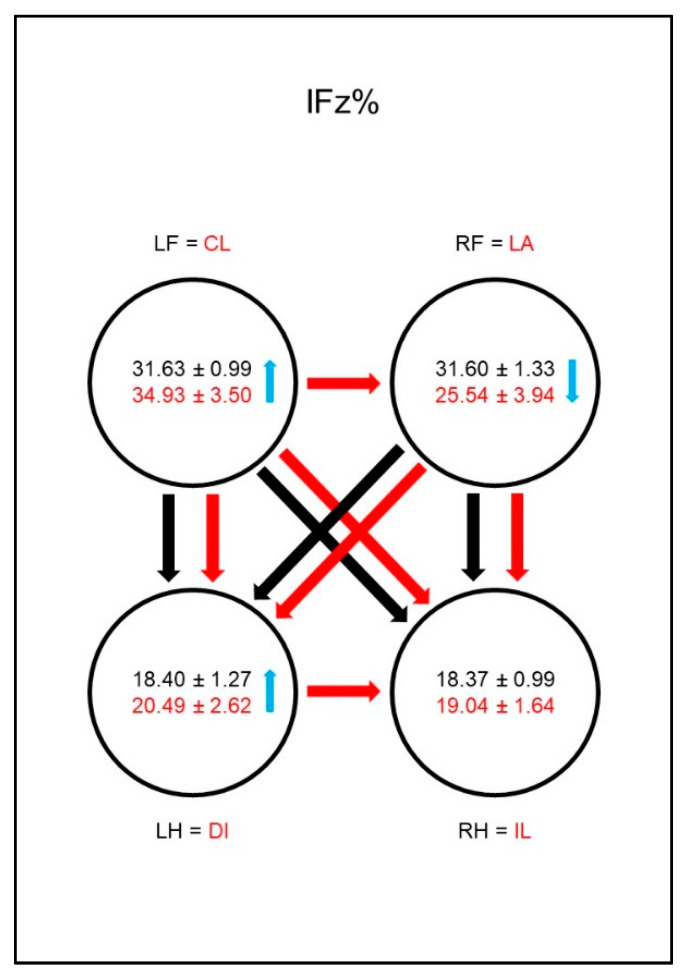
Vertical impulse as a percentage of total force (IFz%) in study group 2 and control group. For detailed explanation and abbreviations refer to Figure 2.

The front limbs showed higher values for PFz% (Figure 11) and IFz% (Figure 12) than the hind limbs in both sound and sick animals. While no differences in these parameters between the contralateral limbs were found in sound animals, in dogs with osteoarthritis of the elbow these values were significantly lower in the lame limb than in the contralateral front limb. This was also evident in the group comparison: the diseased animals showed significantly lower values for PFz% and IFz% of the lame limb than the comparison limb of the sound dogs and, vice versa, higher values in the contralateral front limb. Another compensation mechanism in the diseased dogs was a significantly higher value for PFz% and IFz% in the diagonal hind limb than in the comparison limb of the sound animals. Furthermore, the IFz% was higher in the diagonal hind limb than in the ipsilateral hind limb in the diseased animals.

**Figure 13:**
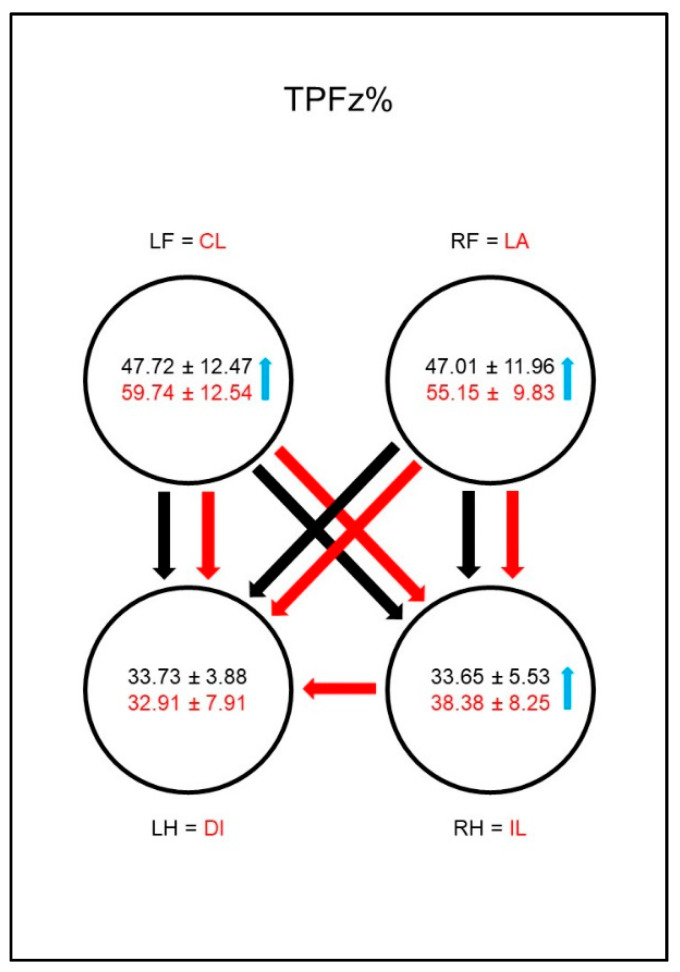
Time to PFz as a percentage of stance phase duration (TPFz%) in study group 2 and control group. For detailed explanation and abbreviations refer to Figure 2.

The PFz occurred earlier (TPFz%, Figure 13) in both groups in both hind limbs than in the front limbs, but while in sound animals there is no difference between the contralateral limb pairs, in diseased animals the PFz occurs significantly later in the ipsilateral hind limb than in the diagonal hind limb. In the group comparison this resulted in a later occurrence of the PFz in the lame limb, contralateral front limb, and ipsilateral hind limb.

**Figure 14:**
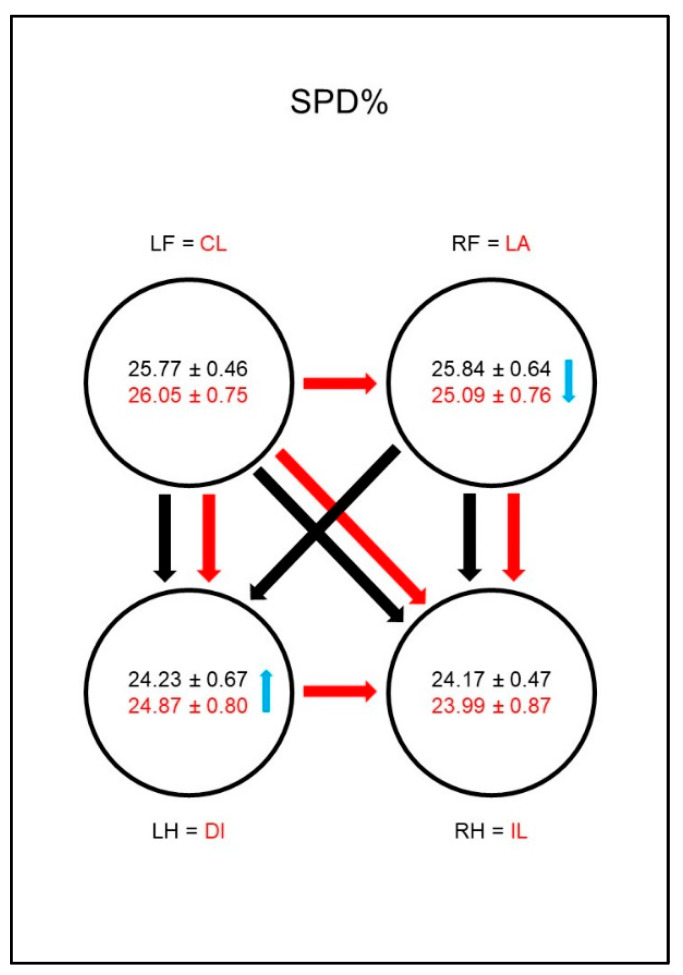
Stance phase duration as a percentage of total stance phase duration (SPD%) in study group 2 and control group. For detailed explanation and abbreviations refer to Figure 2.

The duration of the stance phase (SPD%, Figure 14) was significantly shorter for the hind limbs of sound animals than for the front limbs, and there was no difference between the contralateral limbs in this group. In the diseased animals a significantly shorter SPD% of the lame limb compared to the contralateral front limb was noticeable. In addition, the diagonal hind limb showed a longer SPD% than the ipsilateral hind limb. This led to a disappearance of the significant difference between the lame limb and the diagonal hind limb. In the group comparison, this led to a significantly shortened SPD% in the lame limb compared to the comparison limb of sound dogs and extended SPD% in the diagonal hind limb of diseased animals.

**Figure 15:**
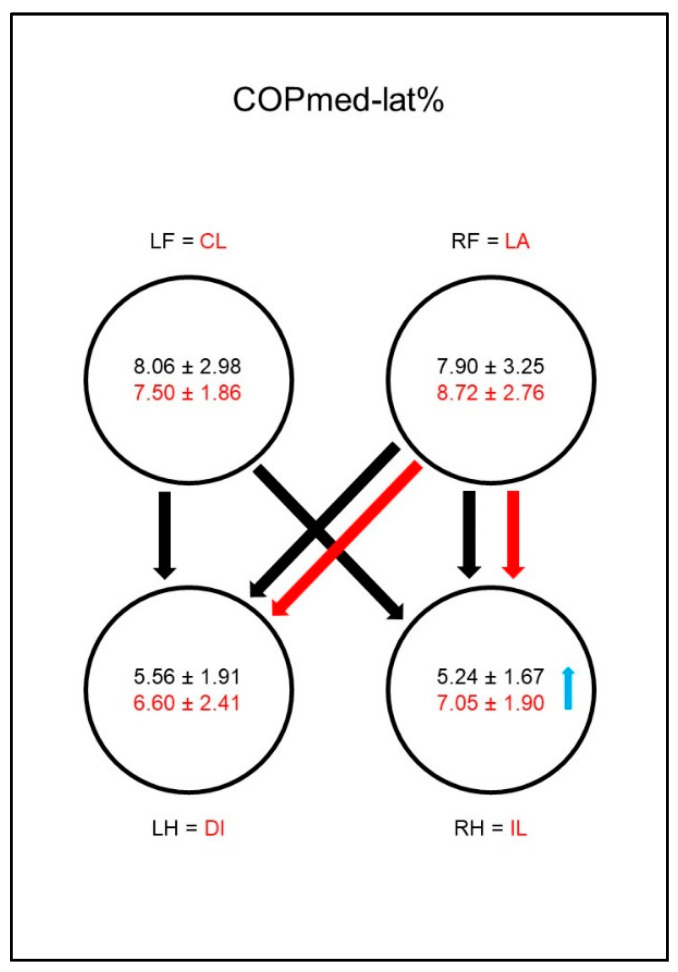
Medio–lateral displacement of the center of pressure as a percentage of maximum width of paw contact area (COPmed-lat%) in study group 2 and control group. For detailed explanation and abbreviations refer to Figure 2.

In sound animals, the mediolateral displacement of the COP (COPmed-lat%, Figure 15) was significantly smaller in both hind limbs than in the front limbs. In the diseased animals, mediolateral COP displacement increased in the ipsilateral hind limb, resulting in a significant difference to the sound animals and also in the disappearance of the significant difference between the contralateral front limb and the ipsilateral hind limb. The significant difference between the contralateral front limb and diagonal hind limb was also lost in the diseased animals, although no significant changes occurred in the respective limbs.

**Figure 16:**
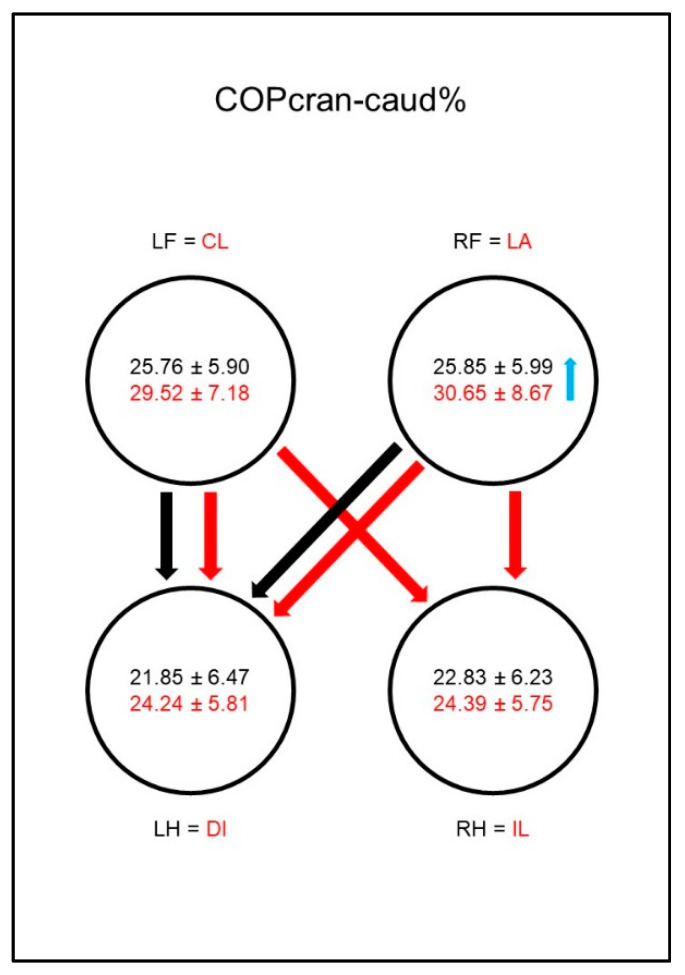
Cranio-caudal displacement of the center of pressure as a percentage of maximum length of paw contact area (COPcran-caud%) in study group 2 and control group. For detailed explanation and abbreviations refer to Figure 2.

Regarding the craniocaudal displacement of the COP (COPcran-caud%, Figure 16), the results were not consistent. In sound animals, the values in the hind limbs were, in general, lower than in the front limbs, but this was only significant when comparing the left hind limb with the front limbs, but not in the right hind limb. In dogs with osteoarthritis of the elbow, a significant increase in the craniocaudal COP displacement in the lame limb was observed in the group comparison. Together with a non-significant increase of this parameter in the contralateral front limb, this resulted in a significant difference between both front limbs and the hind limbs in the diseased dogs.

**Figure 17:**
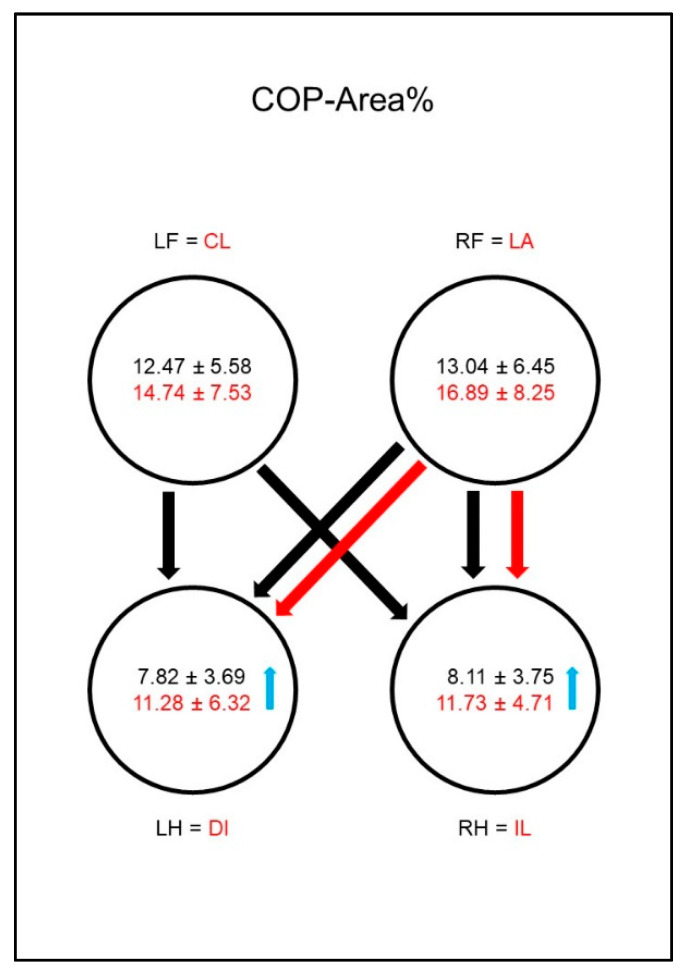
Area encompassed by the boundaries of the center of pressure movement as a percentage of paw contact area (COP-Area%) in study group 2 and control group. For detailed explanation and abbreviations refer to Figure 2.

The COP-Area% (Figure 17) was significantly smaller in the hind limbs of sound animals than in the front limbs. In the diseased dogs, the COP-Area% in both hind limbs increased significantly compared to the sound group, leading to a disappearance of the significant difference between the contralateral front limb and the hind limbs.

**Figure 18:**
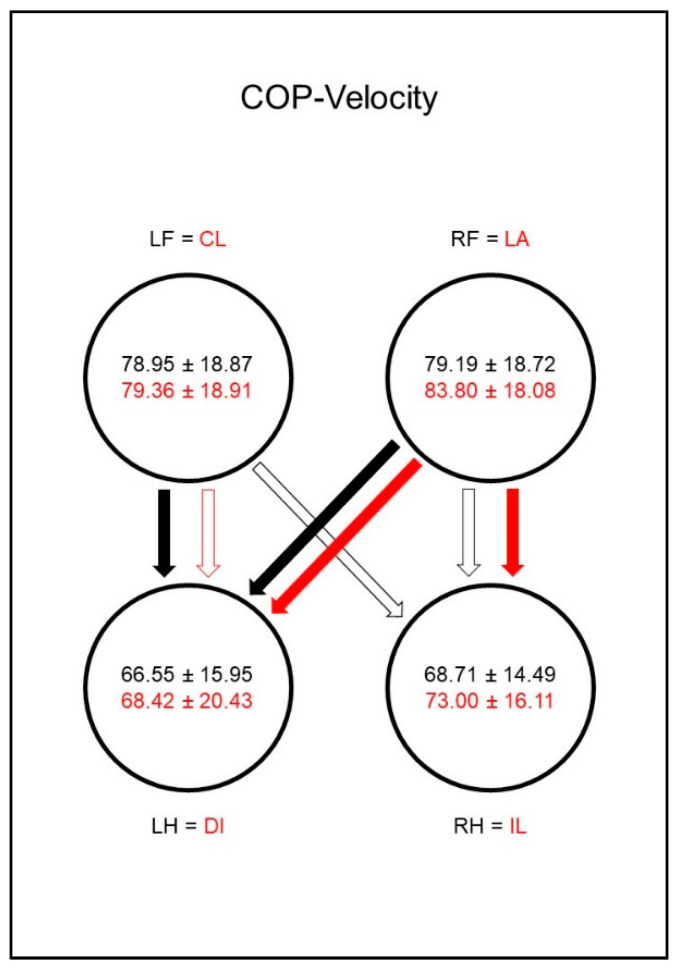
Speed of center of pressure movement in millimeters per second (COP-Velocity, mm/s) in study group 2 and control group. For detailed explanation and abbreviations refer to Figure 2.

The COP-Velocity (mm/s, Figure 18) of sound animals also showed lower values for the hind limbs than for the front limbs, although the significance limit for the right hind limb was narrowly missed. In the diseased animals, a significant difference between the lame limb and both hind limbs was shown. Additionally, a difference just outside the significance limit occurred between the contralateral front limb and the diagonal hind limb.

**Figure 19:**
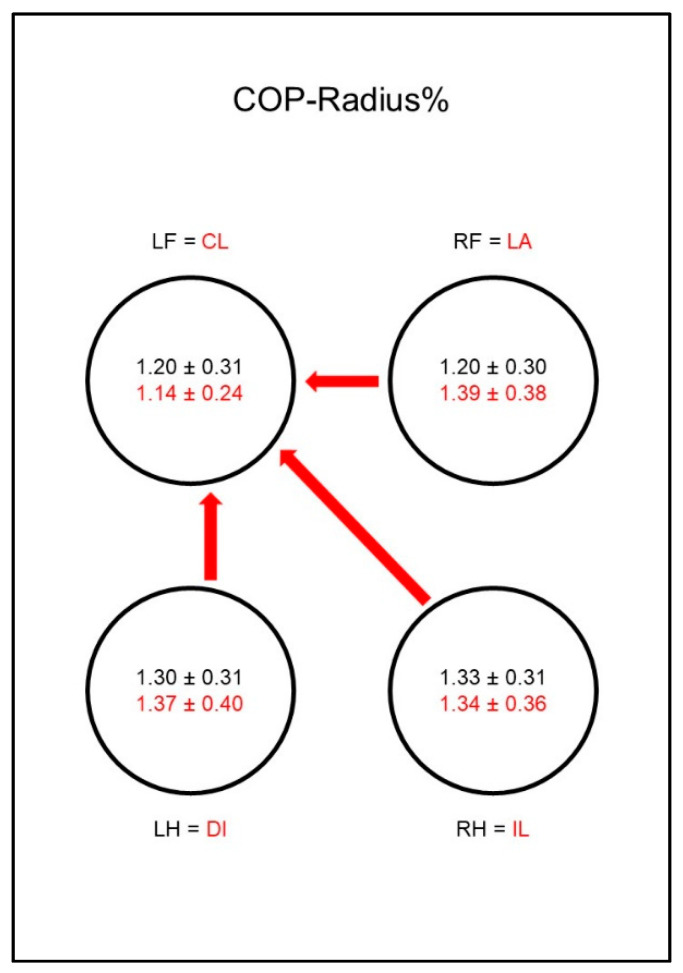
Mean distance of all center of pressure (COP) points to the center point of all COP points as a percentage of paw contact area (COP-Radius%) in study group 2 and control group. For detailed explanation and abbreviations refer to Figure 2.

The situation was different with the COP-Radius% (Figure 19). While in sound animals the radius of the COP did not differ between the limbs, in diseased animals there was a relative reduction in the contralateral front limb with a simultaneous relative increase in the other three limbs. This resulted in a significantly lower radius in the contralateral front limb compared to the other three limbs. However, no difference could be shown between the groups.

An overview of the significant differences in the COP parameters of study group 2 and the control group is displayed in Table 2.

## 4. Discussion

We approached this study with the hypothesis that a stance phase lameness caused by osteoarthritis leads to a compensatory change of the COP-path within the paws, and thus parameters of the COP can be used to depict an osteoarthritis-related lameness. These changes should not only occur within the diseased animals, but also in comparison to a sound control group. The results were according to these expectations and thus clearly confirmed our hypothesis.

In study group 1, the ground reaction forces showed a decreased PFz%, as well as IFz%, and a prolonged TPFz% of the lame limb. In addition to this reduction of the load on the affected limb, a prolonged SPD% of the contralateral limb was found. This suggests that the compensation mechanisms of a hind limb lameness affect the contralateral limb. This has already been shown in the existing literature [29,30]. Additionally, an increased PFz% of the ipsilateral front limb was found in our study. This has already been described by a study that found changes in the load distribution, not only of the contralateral hind limb, but also of both front limbs due to an induced hind limb lameness [31]. That we did not find changes in the contralateral front limb could possibly be explained by the fact that our study did not examine an induced but a chronic lameness. This approach would also explain why no significant changes in the front limbs were found in the study of Rumph et al. [30], where chronic lameness was described.

**Figure 20:**
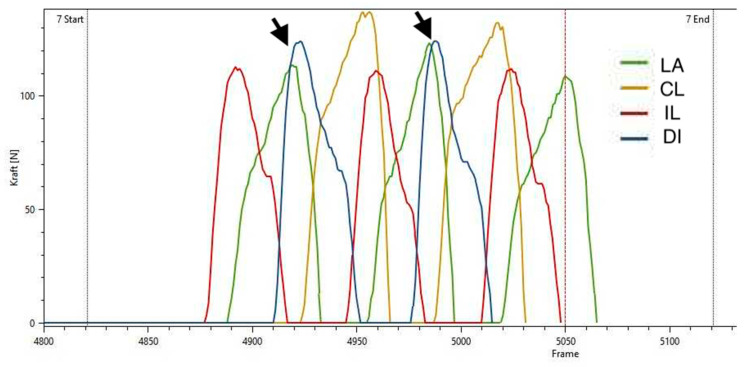
Illustration of the ground reaction forces of a dog from study group 2 as a function of force and time. Each curve corresponds to a contact between a paw and the ground. The arrows mark moments when a reduced load on the lame front limb is superseded by an increased load on the diagonal hind limb. LA = lame limb, CL = contralateral limb, IL = ipsilateral limb, DI = diagonal limb.

Similarly, in study group 2 the ground reaction forces of the lame limb also showed a decreased PFz% and IFz%, as well as a prolonged TPFz%. Additionally, a decreased SPD% was also found in the lame limb. The logical reduction of the load on the lame limb has already been demonstrated by several authors [26,32,33]. Additionally, we found that, in contrast to study group 1, the diseased animals of group 2 showed an increased PFz% and IFz% of the contralateral front limb and the diagonal hindlimb. A study by Bockstahler et al. [33] already described a similar shift to the diagonal hind limb in dogs suffering from osteoarthrosis of the elbow. The results of the contralateral limb, on the other hand, were contradictory because the mentioned study could not show any changes for PFz% or IFz% in comparison to sound animals. The comparison of the limbs within the diseased group, however, showed an increased PFz% and IFz% in the contralateral front limb compared to the lame limb, which is in accordance with our results. Concerning the ipsilateral hind limb, the PFz% and the IFz% remained unaffected in both our and the mentioned study. When comparing these studies, however, it must be noted that different measurement methods were used, as the measurements in the study mentioned above were conducted on a treadmill. A recent study by Braun et al. [26] also investigated the vertical force redistribution in dogs with osteoarthritis of the elbow. To be able to investigate these changes in even more detail, the pads were divided into four quadrants each. In addition to a reduction of the PFz% and IFz% in the lame limb, a redistribution of weight to the caudal area of the contralateral front limb and to the caudomedial area of both hind limbs was found. Additionally, an increase in the caudolateral area of the diagonal hind limb was also found in the IFz%. Again, the changes in the contralateral front limb and the diagonal hind limb show similarities with our results. However, we could not demonstrate the changes in the ipsilateral hind limb with our methods. Another study conducted by Abdelhadi et al. [32] also showed a similar diagonal shift pattern with a decreased PFz and IFz in the lame front limb, increased PFz and IFz in the diagonal hind limb, and unchanged parameters in the ipsilateral hind limb. These repeatedly proven adaptations can be better understood by means of Figure 20. Here, it can be seen that, during the step cycle, the diagonal hind limb is placed on the ground during the stance phase of the lame front limb. Thus, the diagonal hind limb shows an increased load because it takes over the lack of forces of the lame front limb.

In summary, it can be concluded that the compensatory mechanisms of the ground reaction forces and temporospatial parameters are much more complex in dogs with osteoarthritis of the elbow than in dogs with coxarthrosis. In the dogs with osteoarthritis of the hip, compensation occurred mainly via the contralateral hind limb while, apart from an increased PFz% in the ipsilateral front limb, the front limbs showed no changes. In dogs with osteoarthritis of the elbow, on the other hand, compensation was not only provided by the contralateral front limb, but also by the hind limbs, with the diagonal hind limb having higher ground reaction force parameters and an earlier PFz than the ipsilateral limb.

Regarding the COP parameters, the most prominent changes in study group 1 were found in the COPmed-lat% and, as a result, also in the COP-Area%. In the diseased animals, a significant increase of both parameters in the hind limbs could be observed. While in sound dogs both parameters showed significantly lower values in the hind limbs than in the front limbs, almost all of these significant differences were eliminated in the diseased animals.

A proper comparison of these results is difficult as there is little literature on this subject. In the study by Carillo et al. [4], the body COP of dogs suffering from hind limb lameness due to cranial cruciate ligament rupture (CCLR) was investigated. The data were collected in a static way, but despite the drastically different methods, the authors found higher values in the statokinesiogram and stabilogram, and thus a higher COP sway in the diseased dogs compared to the sound control group. Overall, both our results and those of the mentioned study can be interpreted both as a result of the redistribution of body weight as well as an indication that osteoarthritis leads to changes in balance. The latter seems quite possible, considering that in humans osteoarthritis leads to an impaired balance [11]. However, it must be considered that the observation of COP parameters during regular walking is not a direct method to verify postural control. However, the displacement of the COP is an indirect measure of the functionality of postural control and thus a measure of a person’s ability to maintain balance. This should be further investigated in future studies, which directly challenge the postural control of the animal. 

Regarding the changes in the mediolateral displacement of the COP, a possible explanation, especially in the context of hip diseases, can be found in the study of Poy et al. [34]. Here, a greater degree of hip joint adduction and a greater range of abduction–adduction, as well as a greater lateral pelvic movement, was found in dogs with hip dysplasia.

Interestingly, the animals with osteoarthritis of the elbow showed different changes in COP parameters, though some similarities can be found. Similarly to the animals from study group 1, the animals in study group 2 also showed a significant increase of the COP-Area% in both hind limbs. Additionally, we found an increased medio-lateral displacement in the ipsilateral hind limb. As was seen with the changes in the ground reaction forces, an impact of the fore limb lameness on the hind limbs can also be seen here. However, the most prominent change was a significant increase of the craniocaudal COP displacement in the lame limb.

When comparing these results with the outcomes of those of other studies, data on the hind limbs of dogs with front limb lameness are scarce. Concerning the front limbs, however, there are parallels to our own findings. Despite the drastically different methods mentioned above, the study by Carillo et al. [4] found higher values in the statokinesiogram and stabilogram, and thus a higher COP sway in dogs suffering from elbow dysplasia compared to the sound control group. These alterations were even more pronounced in the forelimbs of dogs with elbow dysplasia than in the hindlimbs of dogs suffering from CCLR. 

The study by Manera et al. [24] investigated posturographic changes of standing dogs with osteoarthritis of the elbow. Once again, despite the different methods used, parallels to our results can be found. First, severe changes of the body COP due to the osteoarthritis were found. Even more interestingly, however, the study also provided a graphic illustration of the pressure distribution in the paws including the position of the limb COP. The limb COP of the lame limb shifted craniomedially in the mentioned study. Although no significant change in the mediolateral direction was found in our study, the results are nevertheless in agreement regarding the presence of a change in the craniocaudal direction.

The study by Lopez et al. [16] used dynamic measurements to investigate COP limb path differences of dogs with osteoarthritis due to elbow dysplasia. Despite the similar approach, a comparison with our results is difficult due to different investigated parameters. Nevertheless, the mentioned study showed that differences in COP parameters between sound and lame limbs exist in diseased animals, which is in accordance with our findings. Although in the study of Lopez et al. [16] promising parameters were already used to investigate the COP in the paw of the walking dog, in this paper, we partially used different parameters. Further studies should investigate which parameters (or possible combinations thereof) are best suited to describe compensatory changes of the COP caused by osteoarthritis.

Concerning the significant increase of the COP-Area% in both hind limbs, no reasonable comparisons can be made due to the lack of literature. A possible explanation could be found in the already discussed change of the weight distribution, which also affects the hind limbs. Although in our study the changes in PFz% and IFz% mainly affected the diagonal hind limb, the study of Braun et al. [26] could provide a reasonable explanation. Here, the hind limbs were found to show a redistribution of weight to the caudomedial area of both hind limbs and additionally an increase in the caudolateral area of the diagonal hind limb was found for IFz%. 

However, some limitations of our study must be acknowledged. For example, the dogs with osteoarthritis of the elbow showed a higher asymmetry and thus more lameness than those with coxarthrosis, as well as a significantly higher acceleration than the sound dogs. It is known that the degree of osteoarthritis does not correlate well with changes in the ground reaction forces [35,36], but since the effect of the degree of osteoarthritis was not examined in this study, the question of whether the COP is more sensitive in this respect remains unanswered for the time being.

A further point of criticism is the circumstance that radiographs were only taken of the elbows or hips of the diseased dogs. Even if the inclusion criteria demanded that only elbows or hips were allowed to be painful, this does not mean that there were no other diseases of the musculoskeletal system which could have had at least some influence on the measurement results. This circumstance is primarily explained by the partly retrospective nature of the study. Of course, it would be advantageous to exclude all other diseases, but this is almost impossible in a clinical set-up. For this purpose, not only all joints and the spine would have to be radiographically examined, but also ultrasound scans of frequently affected soft tissue (e.g., biceps tendon) would have to be performed. However, even this would not mean the ultimate exclusion of another disease of the musculoskeletal system. Further, in prospective studies, it is therefore essential to investigate the extent to which competing diseases influence the measured data. Furthermore, it would be of great interest to investigate in further studies whether, for example, diseases of the knee joint lead to other changes than diseases of the hip joint. It should additionally be noted that animals with bilateral disease were also used in our study. Though the less-loaded limb was assumed to be the lame limb, in these animals the contralateral limb might also have been affected by its disease. 

As already discussed in a recent study by Moreira et al. [27], the heterogeneity of the study population is another important limitation to take into account, and a frequent problem in clinical trials. It has already been shown that the ground reaction forces are influenced by breed, body size and weight [37,38,39]. In order to reduce these effects, in our study dogs with similar morphology were chosen if available, but this was only possible to a certain extent. Additionally, most evaluated parameters were normalized (e.g., to total force). However, the high SD of the body mass in all groups indicates that this limitation has to be considered when interpreting the results. The heterogeneity of our population naturally also had an influence on the speed as animals of different sizes and body shapes show great differences in walking gait speed. Nevertheless, the measured velocities were within a narrow range with small standard deviations (1.00 ± 0.15 m/s for study group 1; 0.98 ± 0.19 m/s for study group 2; 1.11 ± 0.21 for the control group) which were comparable to those of Lopez et al. [16]. That the control population in our study was considerably younger than the study populations must also be considered. Both the frequency of potential comorbidities in older animals and the development of dysplasia-induced osteoarthritis over time may have been important factors here. However, in a clinical study it is difficult to find dogs in higher age groups who do not show any musculoskeletal disorders. To confirm our results, the conduct of further studies with a more homogeneous population should be considered.

It should also be noted that the animals in the control group showed no clinical signs of osteoarthritis and had SI% < 3%, but for ethical reasons no radiographs were taken of their joints to classify them as truly free of osteoarthritis. This can, of course, be criticised as the sole orthopaedic examination, as even combined with the assessment of the ground reaction forces, this does not prove that the dogs in the control group did not suffer from some asymptomatic disease of joints and/or other musculoskeletal structures. On the other hand, the question arises whether, even with a radiological examination of all joints and the spine, every orthopaedic disease could have been excluded. Fitzpatrick et al. [40], for example, investigated a cohort of lame dogs and found that some elbows may be classified as normal in the radiographic examination, even though they were clearly found to be affected by joint disease when arthroscopically examined. In addition to a poor correlation between the severity of the disease confirmed by arthroscopy and the signs that are visible in the radiographs, they also found that there was no correlation between the results of these diagnostic methods and the duration of lameness. Overall, however, this results in an interesting question for further research. It should be investigated whether asymptomatic dogs which do show pathologic changes in the joints, show changes in the COP-path. If this is the case, the respective parameters could provide early indications on asymptomatic joint diseases.

In the majority of human studies investigating postural control, data concerning the COP are collected in standing posture. This procedure can sometimes be difficult when dealing with dogs, because dogs with diseases of the musculoskeletal system tend to lie down quickly to save themselves from unnecessary effort and pain. In such cases it can be more practicable to measure dogs during walking, as this can usually be done without problems. Although literature on dynamic measurement of COP in patients with osteoarthritis is still scarce, it is worth investigating this method because, unlike static measurements, this method could actually be also used in everyday clinical practice one day.

A further aspect which should be considered is that the comparison between species is rendered difficult by the fact that the postural control of the bipedal human during walking is solely dependent on the COP within the weight bearing limb while the second limb is in swing phase. This is a major difference compared to the walking dog, where there are always at least two paws touching the ground. If one wishes to compare the results of dogs with those of humans, it is imperative to compare stance measurements.

## 5. Conclusions

Dogs with OA of the elbow or hip show compensatory changes both in the distribution of body mass and in the COP parameters within the paws. The nature of these changes depends on whether a front limb or a hind limb are affected, but similarities could still be found between both investigated diseases. Significant differences can be found not only when comparing the data from the individual limbs of the diseased animals, but also in comparison to the reference limbs of sound animals. In dogs with osteoarthritis of the hip, the primary increase was in the medio-lateral COP displacement and the COP-Area in both hind limbs. In dogs with osteoarthritis of the elbow, however, the medio-lateral COP displacement in the ipsilateral hind limb and the cranio-caudal COP displacement in the lame limb increased. The COP-Area, on the other hand, increased in both hind limbs. The data and findings gathered in this study from both diseased and sound dogs could serve as a base for even more in-depth research on the paw COP, which could enable a diagnostic use in the future.

## Figures and Tables

**Table 1:** Overview of the significant differences in the COP parameters of study group 1 and control group.

Paw		COP Parameter																								
		COPmed-lat%					COPcran-caud%					COP-Area%					COP-Velocity					COP-Radius%				
**Control group**	**LH**	-	a		c			a		c		-	a		c			a		c						
	**RH**	-		b		d						-		b		d			(b)		(d)					
	**LF**		A	B				A					A	B				A	(B)							
	**RF**				C	D				C					C	D				C	(D)					
**Study group 1**	**CL**	+										+												B		
	**LA**	+				d						+											A			
	**DI**																						a	b		
	**IL**					D																				

Within each group significant differences of a parameter between the limbs are marked with corresponding letters, with lower case letters representing the lower value and upper letters representing the higher value (e.g., a/A). For example, in study group 1 there was a significant difference in the COPmed-lat% with the lame limb having a smaller COPmed-lat% (d) than the ipsilateral limb (D). Significant differences of a limb in the group comparison are marked with + and -; with + representing the higher value and - representing the lower value. For example, the lame limb in the study group had a significantly higher (+) COPmed-lat% than the right hind limb of the control group (-). LF = left front limb, RF = right front limb, LH = left hind limb, RH = right hind limb, LA = lame limb, DI = diagonal limb, IL = ipsilateral limb and CL = contralateral limb; COP = Center of pressure; COPmed-lat% = medio–lateral displacement of the COP as a percentage of maximum width of paw contact area; COPcran-caud% = Cranio-caudal displacement of the COP as a percentage of maximum length of paw contact area; COP-Area% = area encompassed by the boundaries of the COP movement as a percentage of paw contact area; COP-Velocity (mm/s) = speed of COP movement in millimeters per second; COP-Radius% = mean distance of all COP points to the center point of all COP points as a percentage of paw contact area.

**Table 2:** Overview of the significant differences in the COP parameters of study group 2 and control group.

Paw		COP Parameter																								
		COPmed-lat%					COPcran-caud%					COP-Area%					COP-Velocity					COP-Radius%				
**Control group**	**LF**		A	B				A					A	B				A	(B)							
	**RF**				C	D	-			C					C	D				C	(D)					
	**LH**		a		c			a		c		-	a		c			a		c						
	**RH**	-		b		d						-		b		d			(b)		(d)					
**Study group 2**	**CL**							A	B									(A)					a	b	c	
	**LA**				C	D	+			C	D				C	D				C	D				C	
	**DI**				c			a		c		+			c			(a)		c			A			
	**IL**	+				d			b		d	+				d					d			B		

Within each group significant differences of a parameter between the limbs are marked with corresponding letters, with lower case letters representing the lower value and upper letters representing the higher value (e.g., a/A). For example, in study group 2 there was a significant difference in the COPmed-lat% with the lame limb having a higher COPmed-lat% (C) than the diagonal limb (c). Significant differences of a limb in the group comparison are marked with + and -; with + representing the higher value and - representing the lower value. For example, the ipsilateral limb in the study group had a significantly higher (+) COPmed-lat% than the right hind limb of the control group (-). For abbreviations refer to Table 1.

## Appendix A

**Table A1:** *p*-values within the study groups and the control group.

Paw			Parameter								
			PFz%	IFz%	TPFz%	SPD%	COPmed-lat%	COPcran-caud%	COP-Area%	COP-Velocity	COP-Radius%
**Control group**	LH	RH	0.79	0.95	0.96	0.75	0.58	0.63	0.81	0.66	0.74
		LF	0.00 *	0.00 *	0.00 *	0.00 *	0.00 *	0.05 *	0.00 *	0.03 *	0.32
		RF	0.00 *	0.00 *	0.00 *	0.00 *	0.01 *	0.05 *	0.00 *	0.03 *	0.30
	RH	LH	0.79	0.95	0.96	0.75	0.58	0.63	0.81	0.66	0.74
		LF	0.00 *	0.00 *	0.00 *	0.00 *	0.00 *	0.14	0.01 *	0.06°	0.19
		RF	0.00 *	0.00 *	0.00 *	0.00 *	0.00 *	0.13	0.01 *	0.06°	0.17
	LF	LH	0.00 *	0.00 *	0.00 *	0.00 *	0.00 *	0.05 *	0.00 *	0.03 *	0.32
		RH	0.00 *	0.00 *	0.00 *	0.00 *	0.00 *	0.14	0.01 *	0.06°	0.19
		RF	0.62	0.92	0.85	0.70	0.88	0.96	0.77	0.97	0.96
	RF	LH	0.00 *	0.00 *	0.00 *	0.00 *	0.01 *	0.05 *	0.00 *	0.03 *	0.30
		RH	0.00 *	0.00 *	0.00 *	0.00 *	0.00 *	0.13	0.01 *	0.06°	0.17
		LF	0.62	0.92	0.85	0.70	0.88	0.96	0.77	0.97	0.96
**Study group 1**	CL	LA	0.00 *	0.00 *	0.07	0.00 *	0.55	0.67	0.75	0.8	0.72
		DI	0.00 *	0.00 *	0.00 *	0.00 *	0.96	0.56	0.89	0.78	0.00 *
		IL	0.00 *	0.00 *	0.00 *	0.02 *	0.07	0.42	0.15	0.13	0.42
	LA	CL	0.00 *	0.00 *	0.07	0.00 *	0.55	0.67	0.75	0.8	0.72
		DI	0.00 *	0.00 *	0.00 *	0.00 *	0.60	0.29	0.61	1.00	0.02 *
		IL	0.00 *	0.00 *	0.00 *	0.00 *	0.04 *	0.19	0.07	0.27	0.33
	DI	CL	0.00 *	0.00 *	0.00 *	0.00 *	0.96	0.56	0.89	0.78	0.00 *
		LA	0.00 *	0.00 *	0.00 *	0.00 *	0.60	0.29	0.61	1.00	0.02 *
		IL	0.65	0.11	0.78	0.08	0.07	0.82	0.16	0.25	0.22
	IL	CL	0.00 *	0.00 *	0.00 *	0.02 *	0.07	0.42	0.15	0.13	0.42
		LA	0.00 *	0.00 *	0.00 *	0.00 *	0.04 *	0.19	0.07	0.27	0.33
		DI	0.65	0.11	0.78	0.08	0.07	0.82	0.16	0.25	0.22
**Study group 2**	CL	LA	0.00 *	0.00 *	0.16	0.00 *	0.08	0.62	0.35	0.41	0.01 *
		DI	0.00 *	0.00 *	0.00 *	0.00 *	0.16	0.01 *	0.09	0.06°	0.02 *
		IL	0.00 *	0.00 *	0.00 *	0.00 *	0.42	0.01 *	0.10	0.22	0.03 *
	LA	CL	0.00 *	0.00 *	0.16	0.00 *	0.08	0.62	0.35	0.41	0.01 *
		DI	0.00 *	0.00 *	0.00 *	0.33	0.01 *	0.00 *	0.01 *	0.01 *	0.86
		IL	0.00 *	0.00 *	0.00 *	0.00 *	0.02 *	0.01 *	0.01 *	0.03 *	0.62
	DI	CL	0.00 *	0.00 *	0.00 *	0.00 *	0.16	0.01 *	0.09	0.06°	0.02 *
		LA	0.00 *	0.00 *	0.00 *	0.33	0.01 *	0.00 *	0.01 *	0.01 *	0.86
		IL	0.09	0.03 *	0.02 *	0.00 *	0.47	0.93	0.78	0.39	0.76
	IL	CL	0.00 *	0.00 *	0.00 *	0.00 *	0.42	0.01 *	0.10	0.22	0.03 *
		LA	0.00 *	0.00 *	0.00 *	0.00 *	0.02 *	0.01 *	0.01 *	0.03 *	0.62
		DI	0.09	0.03 *	0.02 *	0.00 *	0.47	0.93	0.78	0.39	0.76

Significant differences within the groups are marked with *; differences just outside the significance limit are marked with °. For example, in the control group the PFz% showed a significant difference (*) of the left hind limb compared to both left and right front limb. LF = left front limb, RF = right front limb, LH = left hind limb, RH = right hind limb, LA = lame limb, DI = diagonal limb, IL = ipsilateral limb and CL = contralateral limb; PFz% = Peak vertical force as a percentage of total force; IFz% = Vertical impulse as a percentage of total force; TPFz% = Time to PFz as a percentage of stance phase duration; SPD% = Stance phase duration as a percentage of total stance phase duration; COP = Center of pressure; COPmed-lat% = medio–lateral displacement of the COP as a percentage of maximum width of paw contact area; COPcran-caud% = Cranio-caudal displacement of the COP as a percentage of maximum length of paw contact area; COP-Area% = area encompassed by the boundaries of the COP movement as a percentage of paw contact area; COP-Velocity (mm/s) = speed of COP movement in millimeters per second; COP-Radius% = mean distance of all COP points to the center point of all COP points as a percentage of paw contact area.

**Table A2:** *p*-values of the group comparison (study group 1/control group as well as study group 2/control group).

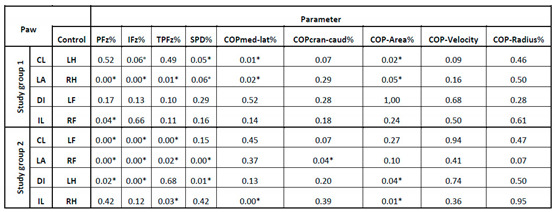

Significant differences between the respective study group and the control group are marked with *; differences just outside the significance limit are marked with °. For example, there was a significant difference (*) between the lame limb of study group 1 compared to the right hind limb of the control group. For abbreviations refer to Table A1.

## Appendix B

**Table A3:** Overview of the significant differences in the parameters of study group 1 and control group.

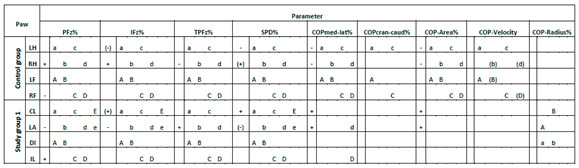

Within each group significant differences of a parameter between the limbs are marked with corresponding letters, with lower case letters representing the lower value and upper letters representing the higher value (e.g., a/A). For example, in study group 1 there was a significant difference in the COPmed-lat% with the lame limb having a smaller COPmed-lat% (d) than the ipsilateral limb (D). Significant differences of a limb in the group comparison are marked with + and -; with + representing the higher value and - representing the lower value. For example, the lame limb in the study group had a significantly higher (+) COPmed-lat% than the right hind limb of the control group (-). For abbreviations refer to Table A1.

**Table A4:** Overview of the significant differences in the parameters of study group 2 and control group.

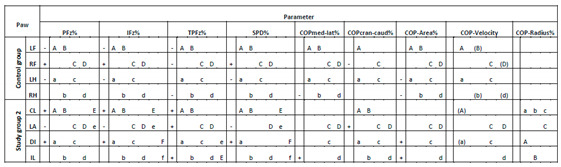

Within each group significant differences of a parameter between the limbs are marked with corresponding letters, with lower case letters representing the lower value and upper letters representing the higher value (e.g., a/A). For example, in study group 1 there was a significant difference in the COPmed-lat% with the lame limb having a smaller COPmed-lat% (d) than the ipsilateral limb (D). Significant differences of a limb in the group comparison are marked with + and -; with + representing the higher value and - representing the lower value. For example, the lame limb in the study group had a significantly higher (+) COPmed-lat% than the right hind limb of the control group (-). For abbreviations refer to Table A1.
